# A Rare Case of Craniopharyngioma in the Temporal Lobe

**DOI:** 10.1155/2017/4973560

**Published:** 2017-12-26

**Authors:** Sasan Razmjoo, Seyed Nematollah Jazayeri, Mohammad Bahadoram, Maedeh Barahman

**Affiliations:** ^1^Department of Clinical Oncology, Ahvaz Jundishapur University of Medical Sciences, Ahvaz, Iran; ^2^Department of Pathology, Ahvaz Jundishapur University of Medical Sciences, Ahvaz, Iran; ^3^Medical Student Research Committee, Faculty of Medicine, Ahvaz Jundishapur University of Medical Sciences, Ahvaz, Iran; ^4^Department of Radiation Oncology and Firoozgar Clinical Research Development Center, Iran University of Medical Science, Tehran, Iran

## Abstract

Herein, we report on a rare case of craniopharyngioma arising in the left temporal lobe with no prior history of head trauma or surgery. There was a solid-cystic mass in the left temporal lobe on MR images. To the best of our knowledge, this is the second case of a craniopharyngioma occurring in the temporal lobe.

## 1. Introduction

Craniopharyngiomas are rare benign epithelial tumors, arising from the pituitary stalk or gland and developing in the sellar and parasellar region of the central nervous system and constitute approximately 3% of all intracranial tumors [1, 2]. These rare tumors are the most common form of nonneuroepithelial neoplasms in pediatrics [3]. Several cases of craniopharyngioma arising from unusual locations other than the sellar and parasellar regions have been described in the literature [3–11]. In this article, we present a rare case of craniopharyngioma arising from the left temporal lobe. Temporal lobe is an extremely unusual location for this tumor and we could find only one case of craniopharyngioma in this location reported in 1999 at Korea [4].

## 2. Case Presentation 

An 18-year-old woman presented with headache, dizziness, and blurred vision in August 2011, with no previous history of head trauma or surgical operation. General physical examination was normal and neurological examination revealed right homonymous hemianopia. All laboratory blood tests were unremarkable.

**Figure 1:**
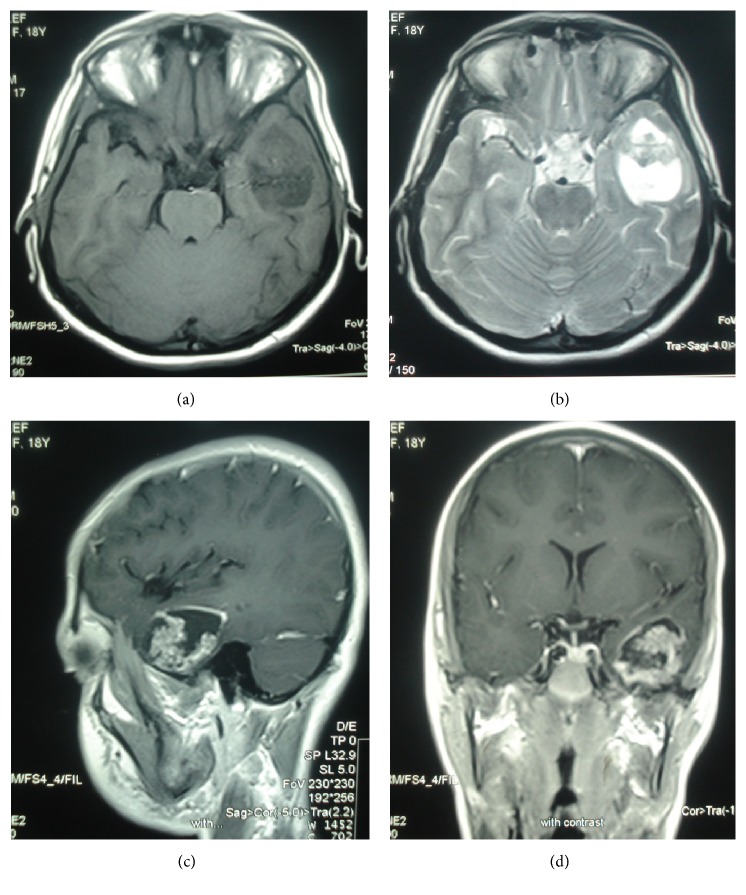
Craniopharyngioma of the left temporal lobe. Axial T1-weighted (a) and T2-weighted (b) MR images show a solid-cystic mass with no significant edema at left temporal lobe. Sagittal (c) and coronal contrast enhanced T1-weighted (d) MR images show peripheral wall enhancement and enhancing solid component.

The magnetic resonance imaging of the brain revealed a large heterogeneous solid-cystic mass in the left temporal lobe with no connection to the craniopharyngeal duct, the suprasellar, or intrasellar regions, although there was no significant edema or mass effect (Figure 1). Contrast enhanced T1-weighted images showed ring enhancement in solid component and peripheral wall (Figure 1).

**Figure 2:**
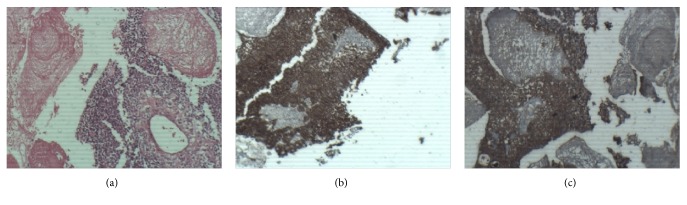
Photomicrograph (a) shows neoplastic tissue composed of solid, pseudopapillary structures lined by several layers of tumoral cells (original magnification, 100; hematoxylin-eosin staining). Photomicrograph (b, c) shows positive cytokeratin and EMA staining in tumoral cells, respectively (original magnification, 100; immunohistochemistry staining).

A few days later, the patient underwent complete surgical resection of the mass. She received 5040 cGy adjuvant radiation therapy in 28 fractions. There was no macroscopic residue in posttreatment contrast enhanced MRI nor local recurrence in follow-up MRI. The histopathologic examination revealed neoplastic tissue composed of solid, pseudopapillary structures lined by several layers of tumoral cells which were cytokeratin and EMA positive in immunohistochemistry study. These data revealed the histologic diagnosis of papillary craniopharyngioma (Figure 2).

## 3. Discussion

There are two histological subtypes of craniopharyngioma: the adamantinous and papillary types [1]. The adamantinomatous variant is much more common than papillary variant (9 : 1) and are pathologically distinct [3]. Squamous-papillary craniopharyngiomas are predominantly solid or mixed solid-cystic masses, which are often observed in adults at suprasellar location. The solid components of the mass usually have an intense and inhomogeneous enhancement with some necrotic areas and rare calcification foci. Adamantinous craniopharyngiomas, on the other hand, are predominantly cystic lobulated masses, which are seen in children at intrasellar/suprasellar location. In 15% of craniopharyngiomas, both adamantinous and squamous-papillary subtypes could be seen [11].

A primary ectopic craniopharyngioma is a rare tumor that diagnoses for the first time without any Rathke's pouch within the vestigial craniopharyngeal duct. Secondary ectopic craniopharyngiomas are rare event. Suspected recurrence of a previously operated cancer arises from tumor cell spillage along the resection corridor or via cerebrospinal fluid dissemination [12].

Our preoperative diagnosis was of low grade glioma because of the enhancing well-defined solid-cystic appearance of the mass with no significant edema or mass effect. Craniopharyngiomas are not usually included in the differential diagnosis of such a mass at left temporal lobe; however, the histopathologic findings showed papillary craniopharyngioma.

There are two theories that may explain the origin of these tumors according to the different histological subtypes, together with the characteristic location of these tumors in the sellar and parasellar region. According to the embryogenetic theory, the adamantinomatous subtype arises from epithelial remnants of the craniopharyngeal duct, which connects the stomodeal ectoderm with the evaginated Rathke's pouch, which in turn forms the adenohypophysis. Rathke's pouch and the craniopharyngeal duct are derived from the stomadeum, which in turn forms teeth primordia. Thus, craniopharyngiomas can potentially arise anywhere along the migration of Rathke's pouch, such as the nasopharynx, paranasal sinuses, third ventricle, and posterior fossa. According to the metaplastic theory, the squamous papillary subtype is a result of metaplasia of squamous epithelial cell rests that are remnants of the part of the stomadeum that contributed to the buccal mucosa [13]. There is no clear embryological evidence for development of craniopharyngiomas at the temporal lobe, and therefore we think that the metaplastic theory is more reasonable than the embryogenetic theory to explain our case.

In conclusion, clinical (age of our case and MR imaging characteristics other than location) and histopathologic findings all were compatible with primary papillary craniopharyngioma, and to the best of our knowledge, this is the second case of a craniopharyngioma originating in the temporal lobe. It is likely to be derived as a result of metaplasia of squamous epithelial cell rests that are remnants of the part of the stomadeum.

## References

[1] Garnett M. R., Puget S., Grill J., Sainte-Rose C. (2007). Craniopharyngioma. *Orphanet Journal of Rare Diseases*.

[2] Van Effenterre R., Boch A. L. (2007). [Craniopharyngiomas]. *Ann Endocrinol*.

[3] Lubuulwa J., Lei T. (2016). Pathological and Topographical Classification of Craniopharyngiomas: a Literature Review. *Journal of Neurological Surgery Reports*.

[4] Sohn C.-H., Baik S. K., Kim S.-P., Kim I.-M., Sevick R. J. (2004). Craniopharyngioma in the temporal lobe: A case report. *Korean Journal of Radiology*.

[5] Khalatbari M. R., Borghei-Razavi H., Samadian M., Moharamzad Y., Schick U. (2012). Isolated primary craniopharyngioma in the cerebellopontine angle. *Journal of Clinical Neuroscience*.

[6] Waga S., Morikawa A., Sakakura M. (1979). Craniopharyngioma with Midbrain Involvement. *JAMA Neurology*.

[7] Fujimoto Y., Matsushita H., Velasco O., Rosemberg S., Plese J. P., Marino R. (2002). Craniopharyngioma involving the infrasellar region: A case report and review of the literature. *Pediatric Neurosurgery*.

[8] Solarski A., Panke E. S., Panke T. W. (1978). Craniopharyngioma in the pineal gland. *Archives of Pathology & Laboratory Medicine*.

[9] Kachhara R., Nair S., Gupta A. K., Radhakrishnan V. V., Bhattacharya R. N. (2002). Infrasellar craniopharyngioma mimicking a clival chordoma: a case report. *Neurol India*.

[10] Gokalp H. Z., Egemen N., Ildan F., Bacaci K. (1991). Craniopharyngioma of the posterior fossa. *Neurosurgery*.

[11] Sartoretti-Schefer S., Wichmann W., Aguzzi A., Valavanis A. (1997). MR differentiation of adamantinous and squamous-papillary craniopharyngiomas. *AJNR. American Journal of Neuroradiology*.

[12] Ortega-Porcayo L. A., Ponce-Gómez J. A., Martínez-Moreno M., Portocarrero-Ortíz L., Tena-Suck M. L., Gómez-Amador J. L. (2015). Primary ectopic frontotemporal craniopharyngioma. *International Journal of Surgery Case Reports*.

[13] Miller D. C. (1994). Pathology of craniopharyngiomas: Clinical import of pathological findings. *Pediatric Neurosurgery*.

